# Establishment and Optimization of Molecular Cytogenetic Techniques (45S rDNA-FISH, GISH, and Fiber-FISH) in Kiwifruit (*Actinidia* Lindl.)

**DOI:** 10.3389/fpls.2022.906168

**Published:** 2022-06-06

**Authors:** Yang Zhao, Honghong Deng, Yao Chen, Jihan Li, Silei Chen, Chunyan Li, Xue Mu, Zhongrong Hu, Kunming Li, Weixing Wang

**Affiliations:** ^1^College of Horticulture and Landscape Architecture, Southwest University, Chongqing, China; ^2^Institute of Pomology and Olericulture, Sichuan Agricultural University, Chengdu, China; ^3^Xinxiang Academy of Agricultural Sciences, Xinxiang, China; ^4^Horticultural Research Institute, Yunnan Academy of Agricultural Sciences, Kunming, China; ^5^Horticultural Research Institute, Guizhou Academy of Agricultural Sciences, Guiyang, China

**Keywords:** 45S rDNA-FISH, blocking DNA, fiber-FISH, GISH, nuclei isolation, post-hybridization washing temperature

## Abstract

The kiwifruit (*Actinidia chinensis*) has long been regarded as “the king of fruits” for its nutritional importance. However, the molecular cytogenetics of kiwifruit has long been hampered because of the large number of basic chromosome (*x* = 29), the inherent small size and highly similar morphology of metaphase chromosomes. Fluorescence *in situ* hybridization (FISH) is an indispensable molecular cytogenetic technique widely used in many plant species. Herein, the effects of post-hybridization washing temperature on FISH, blocking DNA concentration on genomic *in situ* hybridization (GISH), extraction method on nuclei isolation and the incubation time on the DNA fiber quality in kiwifruit were evaluated. The post-hybridization washing in 2 × saline sodium citrate (SSC) solution for 3 × 5 min at 37^°^C ensured high stringency and distinct specific FISH signals in kiwifruit somatic chromosomes. The use of 50 × blocking DNA provided an efficient and reliable means of discriminating between chromosomes derived from in the hybrids of *A. chinensis* var. *chinensis* (2*n* = 2*x* = 58) × *A. eriantha* (2*n* = 2*x* = 58), and inferring the participation of parental genitors. The chopping method established in the present study were found to be very suitable for preparation of leaf nuclei in kiwifruit. A high-quality linear DNA fiber was achieved by an incubation of 20 min. The physical size of 45S rDNA signals was approximately 0.35–0.40 μm revealed by the highly reproducible fiber-FISH procedures established and optimized in this study. The molecular cytogenetic techniques (45S rDNA-FISH, GISH, and high-resolution fiber-FISH) for kiwifruit was for the first time established and optimized in the present study, which is the foundation for the future genomic and evolutionary studies and provides chromosomal characterization for kiwifruit breeding programs.

## Introduction

Kiwifruit belonging to the genus *Actinidia* Lindl. the family Actinidiaceae (Ericales) is one of the most recently domesticated fruit crops (Ferguson, 2016). Despite the short history of domestication, kiwifruit has become a commercially important fruit crop throughout the world with an annual production of approximately 4.3 million tonnes in 2018 (Research and Markets, 2020). At present, China (2.1 million tonnes) is the largest kiwifruit producer, accounting for 50% of the total, followed by Italy (555,000 t) and New Zealand (437,000 t) (Research and Markets, 2020). As the king of fruits, kiwifruit contains a wide range of nutritional compounds, including sugar, organic acids, dietary fiber, minerals, vitamin E, folic acid, antioxidants and phytonutrients, particularly the exceptionally high content of vitamin C (Ma et al., 2017; Richardson et al., 2018; Zehra et al., 2020). As an important horticultural cash crop, the kiwifruit is not only consumed domestically, but also imports from abroad, constituting a globally traded commodity (Ma et al., 2017). The kiwifruit industry has greatly contributed to the global economy by generating over $10 billion (Wu et al., 2019).

Despite substantial study of kiwifruit, little is known about its molecular cytogenetic characteristics. The molecular cytogenetics of *Actinidia* species has long been hampered because of the large number of basic chromosome (*x* = 29), the inherent small size (between 0.6 and 1.5 μm) and highly similar morphology of metaphase chromosomes (Huang and Ferguson, 2007; Ferquson and Huang, 2016). Molecular cytogenetics provides an integrated representation of molecular biology and cytogenetics, and involves the number, structure, function and behavior of mitotic and meiotic chromosomes, chromosome recombination and transmission, and the physical organization of certain DNA sequences (Jones and Moore, 2012). The advent of Fluorescence *in situ* hybridization (FISH) almost 40 years ago (Langer-Safer et al., 1982) marked the beginning of a new era for molecular cytogenetics (Volpi and Bridger, 2008), and has become an indispensable technique for chromosome identification (Xu et al., 2016; Jiang, 2019) and genome sequencing in plant species (Stack et al., 2009).

The advancements in availability of genomic resources and degree of resolution, such as genomic *in situ* hybridization (GISH) and fiber-FISH have widely widened the scope of FISH applications (Xu et al., 2016), which now range from karyotype characterization to integration of genetic linkage maps with chromosomal maps (Jiang, 2019). Using the total genomic DNA as probe and blocking DNA, GISH is a modification of FISH, and it enables to distinguish chromosomes from different genomes in an intact cell (Xu et al., 2016; Ramzan et al., 2017). As a straightforward technique, GISH has been widely applied to the study of chromosomal evaluation, cytogenetical classification, genomic constitution, polyploidy confirmation, hybrid verification, and introgression breeding in horticultural crops (Ramzan et al., 2017). Fiber-FISH allows high-resolution mapping of the repetitive DNA sequences, large and complex genomic loci, and the cloned and organelle DNA molecules on DNA and chromatin fibers (Volpi and Bridger, 2008; Walling et al., 2012). The application of FISH on extended DNA and chromatin fibers allows the physical mapping of individual genes or other small DNA molecules at a resolution of 1–400 kb (Ersfeld, 2004), with 1 kb corresponding to ∼340 nm on a completely relaxed DNA double helix (Heiskanen et al., 1996). The advantage of high resolution of fiber-FISH has thus attracted considerable interest in molecular cytogenetics of different groups of species (Volpi and Bridger, 2008; Xu et al., 2016; Jiang, 2019).

The majority of cytogenetic studies in the genus *Actinidia* have been concentrated on chromosome counts (Huang and Ferguson, 2007; Ferquson and Huang, 2016), ploidy variation determination (Kataoka et al., 2010), and chromosome morphology-based karyotype description (He et al., 2003, 2005). He et al. (2003) reported the diploid *A. chinensis* var. *chinensis* had 29 pairs of homologous chromosomes with 2*n* = 2*x* = 58. Cytogenetically, the genus *Actinidia* presented a structured reticulate pattern of diploid (2*n* = 2*x* = 58), tetraploid (2*n* = 4*x* = 116), hexaploid (2*n* = 6*x* = 174), octoploid (2*n* = 8*x* = 232) and decaploid (2*n* = 10*x* = 290) in a diminishing frequency (Ferquson and Huang, 2016). The karyotype symmetry (2B), evidenced by the presences of 38 metacentric, 18 submetacentric (2SAT), and 2 telocentric chromosomes, is the characteristic of *A. chinensis* var. *chinensis*, what makes the individual identification and molecular cytogenetic study challenging (He et al., 2003, 2005). Knowledge of molecular cytogenetics can undoubtedly help answer many of the biological questions regarding plant genomics, taxonomy, evolution, phylogeny, genetics and molecular biology (Jones and Moore, 2012). However, to date, molecular cytogenetic studies employing FISH performed in *Actinidia* Lindl. are very scarce. Consequently, the application of molecular cytogenetic technique in kiwifruit is of great importance.

In the present study, to establish and optimize the molecular cytogenetic methods for kiwifruit, FISH physical mapping of 45S ribosomal DNA (rDNA) sites was conducted. In addition, genomic identification of the kiwifruit hybrids was performed by GISH. The high-resolution fiber-FISH technique for kiwifruit was also developed and optimized. The establishment and optimization of 45S rDNA-FISH, GISH and fiber-FISH techniques in kiwifruit herein would make the detailed FISH-based karyotypes of *Actinidia* possible and serve as an important molecular cytogenetic basis for future genomic and evolutionary studies and provides chromosomal characterization for kiwifruit breeding program.

## Materials and Methods

### Plant Materials and Genomic DNA Extraction

Seeds of *A. chinensis* cv. ‘Hongyang’ were germinated at room temperature on a moist filter paper in petri dishes. Root tips and fresh young leaves of the seedling of *A. chinensis* cv. ‘Hongyang’ were used as source materials for the mitotic chromosome preparation of FISH and DNA fiber preparation of fiber-FISH, respectively. Root tips for the chromosome preparation of GISH were obtained from germinated seeds derived from the cross-hybridization between *A. chinensis* var. *chinensis* (2*n* = 2*x* = 58) × *A. eriantha* Benth (2*n* = 2*x* = 58). Parental genomic DNA was extracted from fresh young leaves using a DNeasy^®^ Plant Mini Kit (Qiagen, Valencia, CA, United States) following the manufacturer’s instructions. DNA quality (A_260/280_ and A_260/230_ ratios) and concentration were assessed with a spectrophotometer (NanoDrop; Thermo Fisher Scientific, Waltham, MA, United States). Sterile ultrapure water was used to prepare solutions in this study.

### Chromosome Preparation

Mitotic chromosomes were obtained following our previously reported protocol (Deng et al., 2019) with minor modifications. Briefly, the actively growing root tips of approximately 0.5 cm in length were pretreated with 2 mM 8-hydroxyquinoline (Sigma-Aldrich, St. Louis, MO, United States) for 2.5 h in the dark at room temperature (RT), then fixed in a freshly prepared Carnoy’s solution, composed of 75% ethanol and 25% glacial acetic acid, for a minimum of 3.0 h at RT and stored at –20^°^C until further use. Root apices were softened in an enzyme solution consisting of 4.0% (w/v) cellulase and 0.4% (w/v) pectolyase Y-23 for 100 min at 37^°^C, followed by a 20 min hypotonic treatment. The root meristems were squashed in a drop of freshly prepared Carnoy’s solution on a clean pre-chilled slide and dried on a flame. Slides featuring good-quality metaphase chromosomes were kept at –20^°^C until further application of molecular cytogenetic techniques.

### Probe Labeling

Ruiyang Chen (Nankai University, Tianjin, China) is gratefully acknowledged for providing plasmid containing 45S rDNA. Genomic probes were prepared by sonication to 100–500 bp fragments and DNA size was checked using 1% agarose gel. Fragmented DNA (100 ng μL^–1^) and plasmid harboring the 45S rDNA (100 ng μL^–1^) were labeled with digoxigenin-16-dUTP by random-primed labeling method using DIG-High Prime DNA Labeling and Detection Starter Kit II (Roche, Mannheim, Germany) following the instruction manual supplied by the manufacturer. Blocking DNA was prepared by autoclaving the total genomic DNA for 5 min which fragmented it into approximately 200 bp.

### Fluorescence *in situ* Hybridization

Slide with cytological preparations for 45S rDNA-FISH and GISH was firstly dried at 60^°^C in an oven for at a minimum of 3.0 h, treated with 40 μL of RNase solution (100 μg mL^–1^ in 2 × SSC) and incubated in a humidified chamber (37^°^C) for 1.0 h, followed by three times wash in 2 × SSC at RT for 5 min each. The slide was then treated with 0.01% (w/v) pepsin (Sigma-Aldrich) for 10 min at 37^°^C, and washed twice in 1 × PBS at RT for 5 min each. Following this, the slide was immersed in 1% formaldehyde (Sigma-Aldrich) at RT for 5 min, and then rinsed three times in 2 × SSC at RT for 3 min each. The wash steps above and below were performed with a shaker platform set at 150 rpm.

The hybridization mix of FISH (20 μL for one slide), consisting of 10 μL deionized formamide (Sigma-Aldrich), 4 μL 50% dextran sulfate (Sigma-Aldrich), 2 μL 20 × SSC (pH 7.0), 1 μL 10 mg mL^–1^ sperm ssDNA, 2 ng μL^–1^ probe DNA, and double-distilled water (ddH_2_O), were thoroughly mixed, denatured at 100^°^C for 10 min in an Eppendorf Mastercycler (Eppendorf, Westbury, NY, United States) and immediately cooled it on ice for at least 10 min. The chromosomal DNA was denatured by putting slides in 70% (v/v) formamide (in 2 × SSC) solution at 72^°^C for 2 min. The slides were then dehydrated in a series of 70, 95, and 100% ethanol at –20^°^C for 5 min in each solution, followed by air dry. Probe hybridization, signal detection and chromosome photo-documentation were in the same manner as previously reported (Deng et al., 2019). Post-hybridization washes are essential to a FISH protocol to remove the non-specific hybrid signals. Three temperatures (i.e., 35, 37, and 42^°^C) of post-hybridization washes were compared.

### Genomic *in situ* Hybridization

Genomic *in situ* hybridization (GISH) protocol was in accordance with the FISH protocol above with slight modifications in the hybridization mixture, where the probe DNA was replaced by the equal concentration (2 ng μL^–1^) of fragmented genomic DNA of paternal genitor, and the fragmented maternal DNA was added as the blocking DNA. Seven different concentrations of blocking DNA including 0 (0×), 40 (20×), 60 (30×), 80 (40×), 100 (50×), 120 (60×), and 140 (70×) ng μL^–1^ were applied.

### Interphase Nuclei Extraction

Two different leaf nuclei extraction methods by grinding and chopping followed the methods of Jackson et al. (1998) and Li et al. (2005), respectively, with some modifications. Two grams of fresh leaf tissue were pooled together and ground to a fine powder in liquid nitrogen. Then, 80 mg powder were transferred to a clean 2-mL tube with 2 mL of precooled at 4^°^C nuclei isolation buffer containing 10 mM MgSO_4_, 5 mM KCl, 0.5 mM HEPES, 1 mg mL^–1^ DTT, and 0.25% (v/v) Triton X-100 (Sigma-Aldrich). The samples were then gently shaken on ice for a homogenized stage. The resulting suspension were filtered sequentially via 60 and 30-μm mesh nylon membranes, while on an ice-cold metal block. The filtrate was centrifuged at 2,000 *g*n for 2 min at 4^°^C. The supernatant was removed and the nuclei pellet was suspended in two different volumes (30 and 60 μL) of nuclei store solution containing 10 mM MgSO_4_, 5 mM KCl, 0.5 mM HEPES, and 1 mg mL^–1^ DTT. The concentration of nuclei was determined by staining nuclei with 2-(4-amidinophenyl)-1H-indole-6-carboxamidine (DAPI) (1 μg mL^–1^ in 1 × PBS).

Instead of grinding leaves, in the second methods fresh young leaf samples (80 mg) were finely chopped with a clean razor blade in a total of 2 mL cold nuclei isolation buffer to a homogenized stage. The resulting suspension were filtered via 30-μm mesh nylon membranes. The following procedure was in the same manner as described above. The nuclei in storage solution (10 μL) was mixed in an equal volume (10 μL) of DAPI solution, and analyzed using a microscope (BX61; Olympus, Tokyo, Japan) equipped with a Sensys charged-coupled device (CCD) camera (Qimaging Retiga™ SRV Fast 1394, Vancouver, BC, Canada).

### DNA Fiber Extension and Fiber-FISH

Extension of DNA fibers followed the method of Jackson et al. (1998) with some modifications. The nuclei suspension (1 μL) was deposited in a line across one end of poly-L-lysine glass slide (Sigma-Aldrich) and left to air dry for approximately 5 min. Then, 30 μL of lysis buffer (pH 7.0) containing 0.5% (w/v) SDS, 5 mM EDTA, and 100 mM *Tris* was added on the top of the nuclei and incubated at room temperature for 10, 15, 20, 25, and 30 min. DNA fibers were then extended by dragging the suspension of lysed nuclei down the slide slowly and smoothly with the edge of an 18 mm × 18 mm coverslip. The spreading by gravity were not chosen because it can lead to a non-uniform spreading of biers that result in frequent crossing.

Slide was air-dried for approximately 15 min to a “sticky” point, neither wet nor overdried. Slide was fix in Carnoy’s solution for 2 min, dried at 60^°^C for 45 min and then can be used immediately for FISH or stored in 4^°^C for 5–7 days. DNA fiber quality was checked by staining with 5 μg mL^–1^ DAPI at RT for 20 min and briefly rinsing twice in distilled water, followed by air dry. After staining, the effect of incubation time on the DNA fiber quality was observed under epifluorescence microscope. The fiber-FISH procedures are the same as those in regular FISH protocol above.

## Results and Discussion

### Effect of Post-hybridization Washing Temperature on Fluorescence *in situ* Hybridization in Kiwifruit

**FIGURE 1:**
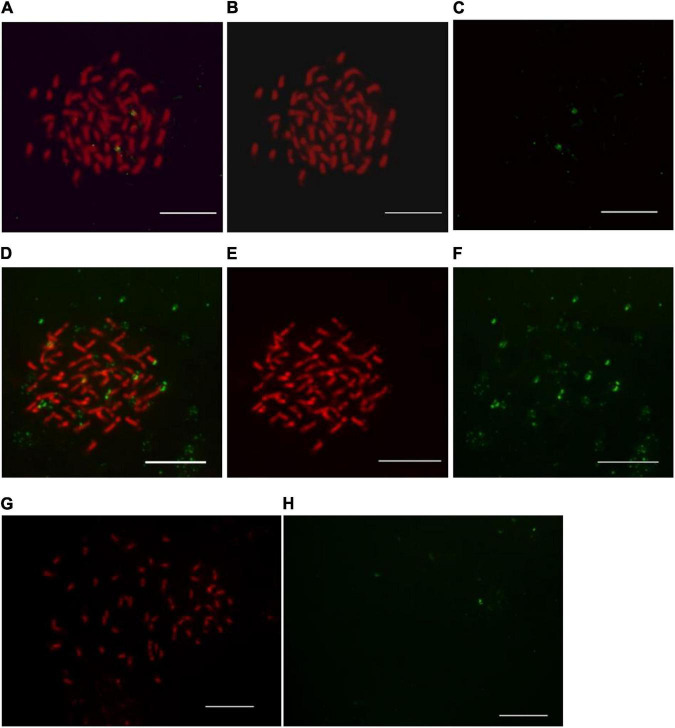
Observation of post-hybridization washing temperature impact on 45S ribosomal DNA-fluorescence *in situ* hybridization in kiwifruit. The post-hybridization washing temperature of 37, 35, and 42^°^C were applied in panels **(A–H)**, respectively. Hybridization sites in panels **(B,C)** and **(E,F)** were digitally separated from the merged images of panel **(A,D)**, respectively. The white scale bar represents 5 μm.

After the probe hybridized to the chromosomal DNA, post-hybridization washing is an essential step of FISH protocol to remove the excess unbound or loosely bound probes and separate the non-specific hybrid signals (Young et al., 2020). Post-hybridization washes associated parameters including temperature, salt and detergent solution concentration can be manipulated to remove non-specific interactions (Young et al., 2020). In this study, we tested three different temperatures (35, 37, and 42^°^C) of post-hybridization washes in a 2 × SSC solution (Figure 1). The salt solution concentration was empirically selected (Deng et al., 2019).

Stringent post-hybridization washing in 2 × SSC solution for 3 × 5 min at 37^°^C ensured the removal of unbound FISH signals and specificity of the detected 45S rDNA signals (Figures 1A–C). Under low post-hybridization washing temperature (35°C), some non-specific FISH signals, excessive background autofluorescence, and some unscorable green signals diminished the visibility of true FISH signals of 45S rDNA (Figures 1D–F). There existed some generalized background on slides but not at chromosome regions (Figures 1D–F). The hybridized probes appeared faint and some of them were even washed away under high post-hybridization washing temperature (42°C) (Figures 1G,H).

Optimization of post-hybridization washing temperature is important for achieving optimal hybridization conditions, though several factors affected the efficiency and quality of hybridization (Bogdanovska-Todorovska et al., 2018). It is recognized that low temperature can lead to inadequate stringency of post-hybridization washing conditions, while high temperature lead to excessive stringency of post-hybridization washing conditions (Zordan, 2011). Thus, the possible reasons for the non-specific FISH signals (Figures 1D–F) and the loss of signals (Figures 1G,H) were assumed to be the insufficient and excessive stringency of post-hybridization conditions, respectively. By evaluating the effects of different post-hybridization washing temperatures on FISH results as presented in Figure 1, we demonstrated that the post-hybridization washing in 2 × SSC solution for 3 × 5 min at 37^°^C ensured high stringency and distinct specific FISH signals in kiwifruit somatic chromosomes.

### Effect of Blocking DNA Concentration on Genomic *in situ* Hybridization in Kiwifruit

Genomic *in situ* hybridization is a variation of FISH and has been widely used to distinguish parental chromosomes or chromosome segments (Xu et al., 2016; Ramzan et al., 2017). The utilization of fragmented genomic DNA as probe and non-target genome as blocking DNA in GISH differentiate it from FISH analysis (Ramzan et al., 2017). Higher plant genomes are composed of high proportion of repetitive DNA families and considered highly conserved in plants (Garrido-Ramos, 2017). Therefore, genome discrimination by GISH often meets considerable complications resulting from the existence of the highly conserved repetitive DNA sequences emerged during the long-term evolution events. GISH works primarily on hybridization of these repetitive DNA sequences (Markova and Vyskot, 2009). In this case a blocking DNA serving as a DNA competitor to avoid the staining of both genomes by the probe DNA is important in hybrids in particular derived from closely related parental genotypes (Markova and Vyskot, 2009).

**FIGURE 2:**
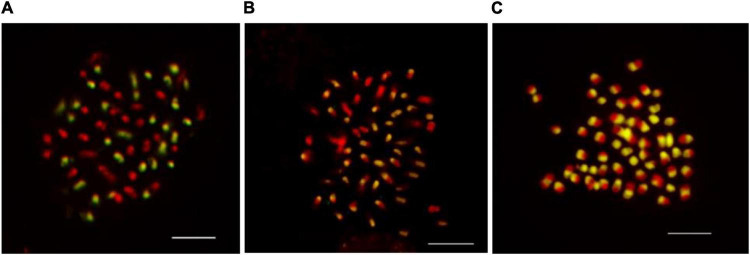
The effect of blocking DNA concentration on genomic *in situ* hybridization results in kiwifruit. The uses 50× **(A)**, 30× **(B)**, and 0× **(C)** more blocking DNA than the probe were compared in this study. The white scale bar represents 5 μm.

In the current study, seven different concentrations of blocking DNA were compared to determine what the ratio of probe/blocking DNA are sufficient to inhibit the chromosome labeling of both parental genomes together. The blocking DNA in a concentration 50 × higher than that of the labeled maternal genomic DNA probe (2 ng μL^–1^) allows efficient hybridization and discrimination of the parental chromosome sets (Figure 2A). The use of lower concentrations (less than 50 × than that of the labeled paternal DNA probe) of the blocking DNA did not exhibit good results and not enable unambiguous differentiation of the two parental chromosome sets (Figure 2B). High concentration (over 50 times) of blocking DNA, however, did not generate hybridization signals, probably because of the high similarities between repetitive DNA sequences that are common in the parental genomes (Marasek et al., 2004; Markova and Vyskot, 2009). The absence of blocking DNA resulted in substantial hybridization sites on whole chromosome sets (Figure 2C), revealing the need of using blocking DNA. Hence, the use of 50 × blocking DNA is an option to GISH protocol for kiwifruit.

Herein, we described an optimal blocking DNA concentration for obtaining reliable and informative GISH results, though the paternal genitors are closely related with morphologically similar and relatively small chromosome sets. In the present study, the GISH technique was first applied to *Actinidia* Lindl. Our results show that GISH will probably provide an efficient and reliable means of discriminating between chromosomes derived from in the hybrids of *A. chinensis* var. *chinensis* (2*n* = 2*x* = 58) × *A. eriantha* Benth (2*n* = 2*x* = 58), and inferring the participation of parental genitors in the karyotypic constitution of in terspecific hybrids in the future.

### An Optimal Fiber-FISH in Physical Mapping of 45S rDNA of *Actinidia* Lindl.

**FIGURE 3:**
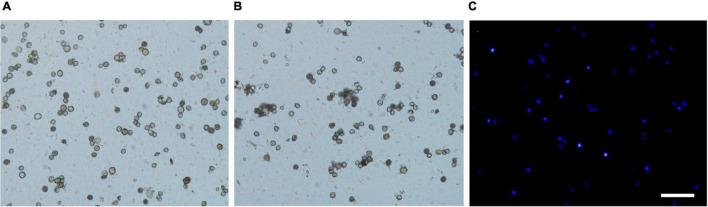
Comparison of nuclei isolation by chopping and grinding methods in kiwifruit. **(A)** Nuclei extracted by chopping method, **(B)** nuclei extracted by grinding method, and **(C)** nuclei extracted by chopping method and stained with 2-(4-amidinophenyl)-1H-indole-6-carboxamidine. The white scale bar represents 100 μm.

FISH performed onto extend DNA fibers released from nuclei has provided a valuable high-resolution tool for physical mapping of up to a few kilobases (Fransz et al., 1996; Valárik et al., 2004; Jiang and Gill, 2006). For example, Fransz et al. (1996) and Jackson et al. (1998) reported the extended DNA fibers of *Arabidopsis thaliana* as 3.27 and 2.87 kb μm^–1^, respectively. Nuclei extraction is one of the most critical aspects of a Fiber-FISH protocol, and it cannot be overstressed that finely extended DNA fiber preparations begin with good nuclei extraction (Li et al., 2005). In the present study, two different methods for isolation of nuclei by directly grinding and chopping fresh leaves in isolation buffer were compared to prepare extended DNA fibers (Figure 3). Fewer nuclei were destroyed using chopping with a blade (Figure 3A) than grinding in liquid nitrogen (Figure 3B). Most of the nuclei extracted by the chopping method remained intact (Figure 3A), while there were more debris presented in the extraction by grinding in liquid nitrogen (Figure 3B). The concentration of nuclei harvested by the chopping method established in the present study reached approximately 5 × 10^6^ to 5 × 10^7^ nuclei/mL (Figure 3C). The chopping method established in the present study were found to be very suitable for preparation of leaf nuclei in kiwifruit.

**FIGURE 4:**
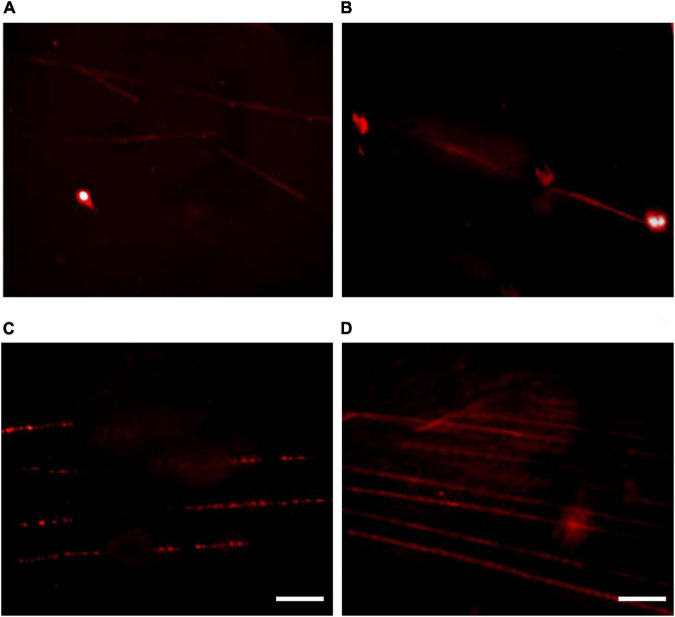
The effect of incubation time on fiber-fluorescence *in situ* hybridization results in kiwifruit. The incubation time of 10 **(A)**, 15 **(B)**, 25 **(C)**, and 20 **(D)** min were compared. The white scale bar represents 5 μm.

A key element in ensuring fiber-FISH data reproducibility is to obtain well-separated/stretched intact DNA fibers (Li et al., 2005). One critical parameter to achieve the best possible stretching/spreading high-quality DNA fibers is the incubation time of nuclei suspension on the glass slide as well as the lysis time (Schwab and Niedzwiedz, 2011; Nieminuszczy et al., 2016). To examine the effects of incubation time on the DNA fiber quality, we tested different incubation times of 10, 15, 20, 25, and 30 min after adding the lysis buffer (Figure 4).

From our experiment it is evident that 10 min of the incubation time after adding the lysis buffer proved to be insufficient to obtain extended DNA fibers (Figure 4A). Increasing the incubation time to 15 min resulted in, to a certain extent, extended DNA fibers, but some of the nuclei have not been fully lysed (Figure 4B). As illustrated in Figure 4C, over 25 min of the incubation time proved to be excessive to obtain extended DNA fiber because the DNA molecules formed a rosary-, like chain of DNA structures with low density. The possible reason for the poor-quality DNA fiber was assumed to be DNA degradation. A high-quality linear DNA molecule was achieved by an incubation of 20 min, and the DNA molecule obtained was straightened and uniformly stretched as presented in Figure 4D.

**FIGURE 5:**
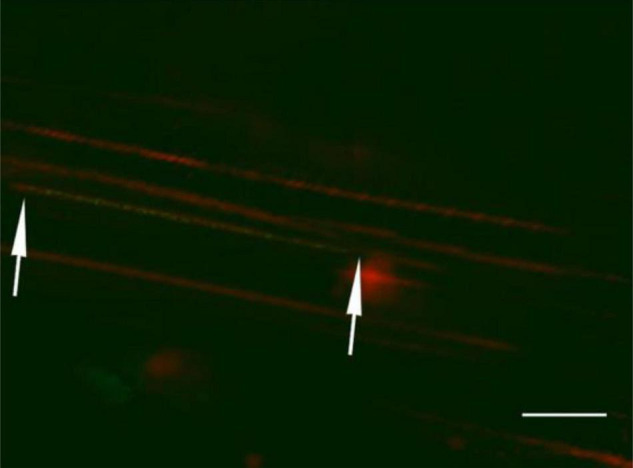
Localization of 45S ribosomal DNA on extended DNA fiber of kiwifruit based on the fiber-fluorescence *in situ* hybridization protocol established and optimized in this study. The white scale bar represents 10 μm.

In addition, FISH analysis for 45S rDNA on extended DNA fibers in kiwifruit was for the first time performed in this study (Figure 5). By applying the highly reproducible fiber-FISH procedures in this study, it was estimated the physical size of 45S rDNA signals was approximately 0.35–0.40 μm (Figure 5). As shown in Figure 5, the 45S rDNA signals appeared as typical beads-on-string pattern of green spots. With the aid of such high-resolution molecular cytogenetic technique established and optimized in the present study, it is possible to accurately chromosome identification, elucidation of evolutionary relationships and delineation of possible chromosomal variations of *Actinidia* Lindl.

To our knowledge, *Actinidia* species have large number of basic chromosomes, relatively small chromosomes with highly similar morphology (Huang and Ferguson, 2007; Ferquson and Huang, 2016); as a result, unequivocal discrimination of individual chromosomes based on traditional cytogenetic methods is rather challenging. Herein, the establishment and optimization of modern molecular cytogenetic techniques (45S rDNA-FISH, GISH, and high-resolution fiber-FISH) will probably provide the necessary means, coupled with other molecular and bioinformatics approaches such as using repetitive DNA sequences as probes, to accurate chromosome identification (currently underway in our laboratory). The outcome presented here are the foundation for the continued fascinating research in the near future.

## Conclusion

The molecular cytogenetics of *Actinidia* species have long been hampered because of the large number of basic chromosome (*x* = 29), the inherent small size and highly similar morphology of metaphase chromosomes. In the present study, the effect of post-hybridization washing temperature on FISH in kiwifruit was evaluated. The post-hybridization washing in 2 × SSC solution for 3 × 5 min at 37^°^C ensured high stringency and distinct specific FISH signals in kiwifruit somatic chromosomes. GISH technique was first applied to *Actinidia* and the use of 50 × blocking DNA provided an efficient and reliable means of discriminating between chromosomes derived from in the hybrids of *A. chinensis* var. *chinensis* (2*n* = 2*x* = 58) × *A. eriantha* Benth (2*n* = 2*x* = 58), and inferring the participation of parental genitors. The chopping method established in the present study were found to be very suitable for preparation of leaf nuclei in kiwifruit. A high-quality linear DNA fiber was achieved by an incubation of 20 min. The physical size of 45S rDNA signals was approximately 0.35–0.40 μm revealed by the highly reproducible fiber-FISH procedures established and optimized in this study. In conclusion, the molecular cytogenetic techniques (45S rDNA-FISH, GISH, and high-resolution fiber-FISH) for kiwifruit established and optimized here are the foundation for the future genomic and evolutionary studies.

## Data Availability Statement

The original contributions presented in the study are included in the article/supplementary material, further inquiries can be directed to the corresponding author.

## Author Contributions

WW and HD conceived and planned the experiment. YZ, HD, and YC performed the whole experiment. JL and SC participated in and performed the 45S rDNA-FISH experiment. CL and XM participated in and performed the GISH experiment. ZH and KL participated in and performed the fiber-FISH experiment. HD wrote and revised the manuscript. All authors read and approved the final manuscript.

## Conflict of Interest

The authors declare that the research was conducted in the absence of any commercial or financial relationships that could be construed as a potential conflict of interest.

## Publisher’s Note

All claims expressed in this article are solely those of the authors and do not necessarily represent those of their affiliated organizations, or those of the publisher, the editors and the reviewers. Any product that may be evaluated in this article, or claim that may be made by its manufacturer, is not guaranteed or endorsed by the publisher.
